# Association Mapping of Ferrous, Zinc, and Aluminum Tolerance at the Seedling Stage in *Indica* Rice using MAGIC Populations

**DOI:** 10.3389/fpls.2017.01822

**Published:** 2017-10-26

**Authors:** Lijun Meng, Baoxiang Wang, Xiangqian Zhao, Kimberly Ponce, Qian Qian, Guoyou Ye

**Affiliations:** ^1^CAAS-IRRI Joint Laboratory for Genomics-Assisted Germplasm Enhancement, Agricultural Genomics Institute in Shenzhen, Chinese Academy of Agricultural Sciences, Shenzhen, China; ^2^Rice Breeding Platform, International Rice Research Institute, Metro Manila, Philippines; ^3^Lianyungang Institute of Agricultural Sciences in Jiangsu Xuhuai Region, Jiangsu Academy of Agricultural Sciences, Lianyungang, China; ^4^Institute of Crop Science and Nuclear Technology Utilization, Zhejiang Academy of Agricultural Sciences, Zhejiang, China

**Keywords:** metal tolerance, seedling stage, MAGIC population, association mapping, rice

## Abstract

Excessive amounts of metal are toxic and severely affect plant growth and development. Understanding the genetic control of metal tolerance is crucial to improve rice resistance to Fe, Zn, and Al toxicity. The multi-parent advanced generation inter-cross (MAGIC) populations were genotyped using a 55 K rice SNP array and screened at the seedling stage for Fe, Zn, and Al toxicity using a hydroponics system. Association analysis was conducted by implementing a mixed linear model (MLM) for each of the five MAGIC populations double cross DC1 (founders were SAGC-08, HHZ5-SAL9-Y3-Y1, BP1976B-2-3-7-TB-1-1, PR33282-B-8-1-1-1-1-1), double cross DC2 (founders of double cross were FFZ1, CT 16658-5-2-2SR-2-3-6MP, IR 68, IR 02A127), eight parents population 8way (founders were SAGC-08, HHZ5-SAL9-Y3-Y1, BP1976B-2-3-7-TB-1-1, PR33282-B-8-1-1-1-1-1, FFZ1, CT 16658-5-2-2SR-2-3-6MP, IR 68, IR 02A127), DC12 (DC1+DC2) and rice multi-parent recombinant inbred line population RMPRIL (DC1+DC2+8way). A total of 21, 30, and 21 QTL were identified for Fe, Zn, and Al toxicity tolerance, respectively. For multi tolerance (MT) as Fe, Zn, and Al tolerance-related traits, three genomic regions, MT1.1 (chr.1: 35.4–36.3 Mb), MT1.2 (chr.1: 35.4–36.3 Mb), and MT3.2 (chr.3: 35.4-36.2 Mb) harbored QTL. The chromosomal regions MT2.1 (chr.2: 2.4–2.8 Mb), MT2.2 (chr.2: 24.5–25.8 Mb), MT4 (chr.4: 1.2 Mb Mb), MT8.1 (chr.8: 0.7–0.9 Mb), and MT8.2 (chr.8: 2.2–2.4 Mb) harbored QTL for Fe and Zn tolerance, while MT2.3 (chr.2: 30.5–31.6 Mb), MT3.1 (chr.3: 12.5–12.8 Mb), and MT6 (chr.6: 2.0–3.0 Mb) possessed QTL for Al and Zn tolerance. The chromosomal region MT9.1 (chr.9: 14.2–14.7 Mb) possessed QTL for Fe and Al tolerance. A total of 11 QTL were detected across different MAGIC populations and 12 clustered regions were detected under different metal conditions, suggesting that these genomic regions might constitute valuable regions for further marker-assisted selection (MAS) in breeding programs.

## Introduction

Trace amounts of metal ions are essential for plant growth and development; however high concentrations result in perturbations in physiological processes, and ultimately productivity. Rice is among the cereal crop able to accumulate high levels of metal, including Ferrous (Fe), Aluminum (Al), and Zinc (Zn). These metals are naturally present at very low levels in paddy soils, but long-term use of chemical fertilizers result to acidity and increased concentration of phytotoxic ion form. Low soil pH (<5.0) favors the production of soluble phytotoxic Al^3+^ ion easily taken up by the root system inhibiting cell division. These results to poor root growth and therefore low ion and water uptake (Panda et al., 2009; Tanaka and Navasero, 2012). Moreover, the anaerobic nature and low redox potential of paddy soils results to the reduction of Fe to Fe^2+^, another soluble ion easily taken up by plants. Excessive amount of Fe^2+^ catalyze the formation of reactive oxygen species (ROS) causing irreversible damage in the cells (Becker and Asch, 2005). Zn is taken up by plant in the form of Zn^2+^ ion during early stages of the plant, which is highly phytotoxic. It was reported that Zn^2+^ has a key role in photosynthetic system. Specifically, it interferes in the photochemical reaction of chloroplast as proven by chlorophyll degradation in lichens, thereby decreasing its photosynthetic activity (Rout and Das, 2003).

Remediation strategies for metal contaminated paddy fields via chemical, physical, or biological means are necessary. However, the available methods are not effective or practical to use due to high input and running costs as well as low efficiency. Breeding new varieties with low metal accumulation in the grain constitutes a cost effective and efficient method to reduce the risk of low rice productivity and improve food safety. Understanding the genetics of metal tolerance is crucial to developing metal tolerance rice varieties.

Quantitative Trait Loci (QTL) mapping is an effective means of dissecting the genetic factors underlying agronomic traits such as metal tolerance. A number of studies have reported QTL for tolerance to a variety of metal. With regards to Fe toxicity in rice, a total of 197 QTL have been reported (Wu et al., 1997, 1998, 2014; Wan J. L. et al., 2003a; Wan J. M. et al., 2003b; Wan et al., 2005; Shimizu et al., 2005; Ouyang et al., 2007; Dufey et al., 2009, 2012, 2015; Fukuda et al., 2012; Zhang et al., 2013; Zhao et al., 2013; Matthus et al., 2015; Ruengphayak et al., 2015; Liu et al., 2016). A total of four chromosomal regions (CR), including CR1 on chromosome 1 between markers RM246 and RM443; CR2 on chromosome 2 between markers RM526 and R758; CR3 on chromosome 3 between markers C515 and C25; and CR4 on chromosome 7 between markers R1245 and RM429, have been found to be involved in the resistance of rice to Fe toxicity (Dufey et al., 2014). However, no genes have yet been cloned. A total of 148 QTL were identified for Al tolerance in rice via linkage mapping using biparental crosses (Wu et al., 2000; Nguyen et al., 2001, 2002, 2003; Ma et al., 2002; Mao et al., 2004; Xue et al., 2006a,b, 2007) and association mapping using natural populations (Famoso et al., 2011; Zhang et al., 2016). The association mapping of 383 rice accessions and linkage mapping populations of recombinant inbred lines (RIL) derived from IR64/Azucena and backcross inbred lines (BIL) derived from Nipponbare/Kasalath//Nipponbare were reported by Famoso et al. (2011). Al-tolerant QTL (*Alt*_*TRG*_*12.1*) encompassing the ART1 locus on chromosome 12 exhibited a large effect (LOD = 7.85, *R*^2^ = 19.3%) in a RIL population. Moreover, three regions corresponding to induced Al-sensitive rice mutants (*ART1, STAR2*, and *Nrat1*) were identified through biparental QTL mapping (Famoso et al., 2011). Association mapping for relative root elongation (RRE) was performed using a core collection of 150 accessions of rice landraces with the highest phenotypic variation (*R*^2^) explained by significant associations of 20.03% (for PSM365) at 21.4 Mb on chromosome 11 (Zhang et al., 2016). A transcription factor, *ART1* (Al resistance transcription factor 1), has been identified for Al tolerance in rice. *ART1* regulates the internal and external detoxification of Al by affecting at least 30 genes (Yamaji et al., 2009; Ma et al., 2014). It is clear that the exposure of the roots to Al triggers both the induction and expression of many Al-resistance genes in rice including *OsNrat1, OsSTAR1/2, OsALS1*, and *OsFRDL4* (Huang et al., 2009, 2012; Xia et al., 2010; Yokosho et al., 2011). Three studies have reported 53 QTL for Zn tolerance using RIL populations (Dong et al., 2006; Zhang et al., 2013; Liu et al., 2016). Among of them, the major QTL *qZNT-1* at interval marker XNpb93-C3029C on chromosome 1 explained 21.9% of the variance (Dong et al., 2006). Four QTL were detected using two independent backgrounds in reciprocal introgression populations, with *QSh2b, QSh7*, and *QSdw5* simultaneously identified in both Teqing-ILs and Lemont-ILs backgrounds (Zhang et al., 2013), while *qZRRDW3* was detected in both MH63-ILs and 02428-ILs backgrounds (Liu et al., 2016). The expression of these QTL in different genetic backgrounds suggests that they might be widespread. Two QTL, *QSdw5* at 17.3–19.5 Mb on chromosome 5 (Zhang et al., 2013) and *qFRSDW11* at C11S49-C11S60 on chromosome 11 (Liu et al., 2016) were expressed under Fe and Zn stress, suggesting that there is a genetic overlap in Fe and Zn toxicity tolerance at the seedling stage.

The genetic architecture of tolerance to Fe, Zn, and Al toxicity in rice appears complex and not yet fully understood. No major locus has been identified, fine-mapped, or cloned thus far. Two limitations of previous studies include that biparental populations cover only a small genetic variability and a small number of genetic markers, resulting in the detection of only a few QTL confined to few genetic backgrounds (Matthus et al., 2015). The use of MAGIC populations with high allelic and phenotypic diversity, in combination with high-density genotyping is an effective means of increasing the genetic mapping resolution for metal tolerance.

Effective phenotyping techniques are a prerequisite in QTL mapping for metal tolerance. Artificial hydroponic systems provide an effective method for metal tolerance screening, as environmental factors such as temperature and acidity of the culture solution are highly controlled (Marmiroli et al., 2011). Moreover, growth rate, leaf color, and the extent of plant injury are common phenotypic traits used to measure the metal tolerance at the seedling stage in rice. The greatest indicator of plant sensitivity to Fe and Zn toxicity is related to leaf symptoms (Audebert and Fofana, 2009). Leaf discoloration and the leaf bronzing index (LBI) are both used to measure the extent of Zn toxicity (Dong et al., 2006), whereas the LBI is used for Fe toxicity (Dufey et al., 2009).

In this study, we aimed to illuminate the genetic basis of tolerance to Fe, Zn, and Al toxicity at the seedling stage by screening highly diverse MAGIC populations in a hydroponic system and employing a 50 K single nucleotide polymorphism (SNP) array. A genome-wide association study (GWAS) was conducted using mixed linear models (MLMs) to determine the loci associated with metal tolerance. The results will be valuable in gene cloning and marker assisted selection (MAS)-based breeding for metal tolerance.

## Materials and methods

### Plant materials

Five MAGIC populations were used in this study. DC1 derived from SAGC-08 (A), HHZ5-SAL9-Y3-Y1 (B), BP1976B-2-3-7-TB-1-1 (C), PR33282-B-8-1-1-1-1-1 (D) parents. DC2 derived from FFZ1 (E), CT 16658-5-2-2SR-2-3-6MP (F), IR 68 (G), IR 02A127 (H) parents. 8way derived from SAGC-08 (A), HHZ5-SAL9-Y3-Y1 (B), BP1976B-2-3-7-TB-1-1 (C), PR33282-B-8-1-1-1-1-1 (D), FFZ1 (E), CT 16658-5-2-2SR-2-3-6MP (F), IR 68 (G), IR 02A127 (H). DC12 was combined use of DC1 and DC2 populations. RMPRIL was combined use of DC1, DC2 and 8-way populations. The eight founders were pairwise crossed to produce four two-way hybrids. These four two-way hybrids were intercrossed in a diallel fashion leading to six 4-way crosses. Two of the 4-way crosses, ABCD and EFGH, were advanced by single seed decent (SSD) to develop the DC1 and DC2 RILs populations. An 8-way cross was made by intercrossing 100 F_1_ plants of the 4-way cross ABCD and 100 F_1_ plants of the 4-way cross EFGH. One thousand 8-way cross F_1_ plants were advanced by SSD to develop the 8-way RILs population. Eight parents showing obvious differences in the agronomic traits and biotic/abiotic stresses (Table 1) were used to develop the population as described by Meng et al. (2016a). A total of 218, 210, 445, 428, and 873 RIL lines of the 4way DC1, 4way DC2, 8way, DC12 and RMPRIL populations were used, respectively.

**Table 1:** Description of the eight parental lines used for developing the MAGIC populations.

**Parents**	**Code**	**Origin**	**Agronomic relevance**	**Resistance relevance**
SAGC-08	A	China	Short grain, thickness stem	Fe, Al, drought tolerance
HHZ 5-SAL 9-Y 3-Y 1	B	IRRI-GSR	Long grain, good grain quality	Salt and Fe tolerance
BP1976B-2-3-7-TB-1-1	C	Indonesia	Long grain	Zn tolerance, blast disease resistance
PR 33282-B-8-1-1-1-1-1	D	Phil Rice	Long grain, high yielding	Al tolerance, bacterial blight resistance
FFZ1	E	China	Long grain, high yielding, large grain, good grain quality	
CT 16658-5-2-2SR-2-3-6MP	F	CIAT	Long grain, high yielding	Bacterial blight resistance
IR 68	G	IRRI	Long grain, large grain, thickness stem	Zn bacterial blight resistance
IR 02A127	H	IRRI	Long grain	Comprehensive disease resistance

### Metals tolerance screening

The screening for Al experiment was conducted under 200 μmol AlCl_3_ and control conditions from July 15 in 2015 in a greenhouse with the day/night temperature of 30/25°C at CAAS, Beijing, China. Rice seeds of 873 MAGIC lines and 8 parents were surface sterilized with 5% sodium hypochlorite, rinsed with water, pre-germinated for 48 h under dark condition and temperature of 30°C. Experiment was conducted using randomized complete block design (RCBD) with two replicates: for one replicate 24 germinated uniform seeds of each line were randomly sown in 96 wells PCR plate (8 × 12). One line was sown in three different plates (8 × 3) that plates have perforated wells at the bottom to facilitate the roots to fully contact with the standard rice nutrient solution (Yoshida et al., 1976). The screening for Fe and Zn experiments were conducted under 300 mg/L Fe^2+^ (as FeSO_4_ × 7H_2_O), 300 mg/L ZnSO_4_ and control conditions from September 10 in 2014 in a greenhouse (NG04-02) with the day/night temperature being about 30/25°C at IRRI (International Rice Research Institute), Laguna, Philippines. Rice seeds including 8 parents and 873 RILs were soaked in demineralized water and germinated at 30°C in the dark for 48 h. Subsequently, 56 uniform seeds per line were directly sown into perforated Styrofoam sheets covered with nylon net at the bottom placed on standard rice nutrient solution. Each hole contained two seeds of the same line or parent. An Augmented RCBD was adopted with six sets of incomplete blocks 8 parents being replicated six times. The 10 days old plants were exposed to Fe, Zn, and Al stress for further 20 days. The pH of the control and all treated groups were adjusted to 4.5 using 1 N NaOH or 1 N HCl, pH were maintained every day and solutions were changed after every 5 days.

The metal tolerance scores (MTS) were measured at 20 days after treatment according to the modified standard evaluation system (Dufey et al., 2012). The MTS for Fe and Zn indicates the severity of toxicity: 1 (Highly tolerance: Normal growth with no leaf symptoms), 3 (Tolerance: Nearly normal growth, but leaf tips or few leaves whitish and rolled), 5 (Moderately tolerant: Growth severely retarded; most leaves rolled; only a few are elongating), 7 (Susceptible: Complete cessation of growth; most leaves dry; some plants dying) and 9 (Highly susceptible: Almost all plants dead or dying).

Three plants of each line from each replicate were harvested for measuring the shoot length (SL) and root length (RL). The roots and shoots were put in craft paper envelop and dried at 60°C for 72 h in an oven. The shoot dry weight (SDW) and root dry weight (RDW) were determined. The indexes of toxicity tolerance, relative shoot length (RSL), relative root length (RRL), relative shoot dry weight (RSDW) and relative root dry weight (RRDW) were calculated according to the following formula: relative trait value (%) = (trait value in treatment)/(trait value in control) × 100.

### Genotyping with the 55 K SNP array

A total of 873 RILs from the MAGIC population, plus the eight parents were genotyped. Approximately 200 ng of DNA from each inbred line was used for genotyping using the Affymetrix® GeneTitan® platform with the Affymetrix® Axiom® Rice Genotyping Array, conducted by the CapitalBio Technology (Beijing, China) according to the manufacturer's protocol. The raw signal CEL files were processed using the Axiom® genotyping best practices. A total of 881 plates passed Dish Quality Control (QC). The probe QC was then determined using samples that passed the QC and were classified into six major categories including “Poly High Resolution,” “Mono High Resolution,” “No Minor Homozygote,” “Off Target Variant,” “Call Rate Below Threshold,” and “Other.” Finally, 39,066 high-quality (Poly High Resolution) SNPs displaying genetic diversity were selected from the common SNPs. In addition, a three-step filtering strategy was applied to select high quality SNPs for QTL mapping. Firstly, markers that were non-polymorphic among the parents were removed. Secondly, all heterozygous genotypes were set as “missing” and markers with more than 10% missing values were removed. Finally, markers with a minor allele frequency (MAF) less than 3% were removed. The number of markers remaining were 22,160, 22,020, 28,505, 28,540, and 28,531 for the DC1, DC2, 8way, DC12 (DC1+DC2), and RMPRIL (DC1+DC2+8way) populations, respectively (Supplementary Table S1).

### Statistical analysis

Adjusted trait value for each RIL was obtained using the PBTools developed by IRRI (http://bbi.irri.org/). Association analysis was conducted using MLM implemented in TASSEL v5.2 (Bradbury et al., 2007). The model uses PCA and kinship to control population structure and the familiar relationship. Bonferroni-corrected threshold probability based on individual tests was calculated to correct for multiple comparisons, using 1/N, where N is the number of individual trait-SNP combinations tested. Significant marker trait associations (MTAs) were identified based on probability level of 1.0 × 10^−4^. Peaks exhibiting significance threshold level within a physical distance of 1.0 Mb were delineated into a single QTL. A QTL explaining more than 10% of the phenotypic variation was considered a major QTL. QTLs detected for different metal stresses with an overlapping confidence interval of 1.0 Mb were defined as a QTL cluster. The R-qqman package was used for creating the Manhattan plot (Turner, 2014). All other statistical analyses were conducted in R 3.3.1 (R Development Core Team, 2016).

## Results

### Phenotypic variation

**Figure 1:**
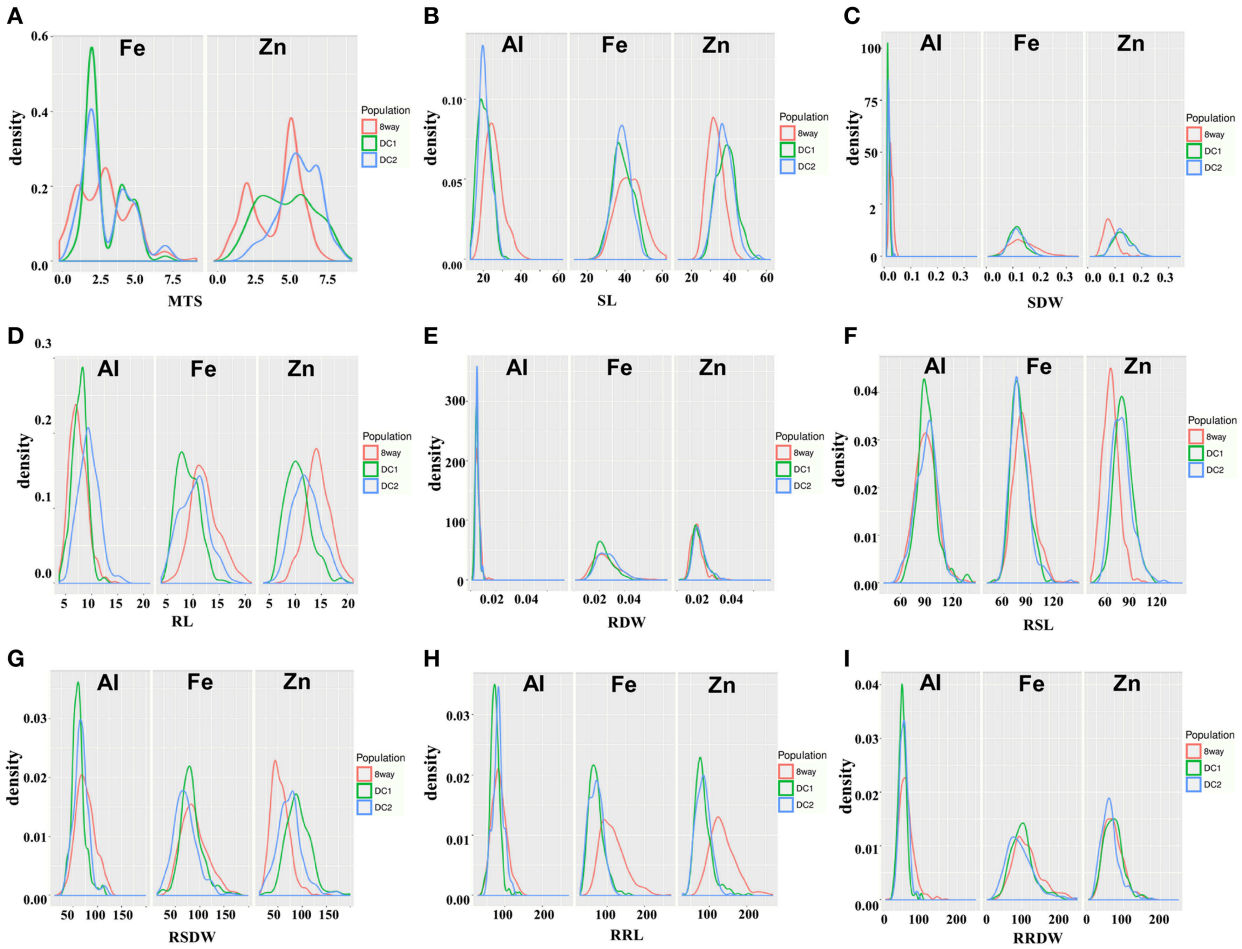
Distribution of nine traits in the DC1, DC2, and 8way populations measured under Fe, Zn, and Al stress conditions. **(A)** MTS, Metal tolerance scores. **(B)** SL, Shoot length. **(C)** RL, Root length. **(D)** SDW, Shoot dry weight. **(E)** RDW, Root dry weight. **(F)** RSL, Relative shoot length. **(G)** RRL, Relative root length. **(H)** RSDW, Relative shoot dry weight. **(I)** RRDW, Relative root dry weight.

All the parental lines differed significantly for MTS under Fe and Zn stress (Table 2). For Fe, the parental lines A (1.0) and B (2.0) had the lowest MTS, while G (4.5) and H (5.0) had the highest MTS among the eight parental lines. For Zn, the parental lines C (3.0) and G (3.0) were the most tolerant, while the parental line B (6.5) was the most sensitive. Transgressive segregations were observed in both directions for all the populations (Figure 1), though the mean scores were not significantly different among populations (Table 2). For Fe, the average values of the DC1 population were 77.8, 70.2, 82.4, and 100.9 for RSL, RRL, RSDW, and RRDW, respectively; for the DC2 population these were 77.8, 68.7, 70.2, and 95.2%; and those of the 8way population were 82.5, 114.7, 88.4, and 113.7. For Zn, the average values of RSL, RRL, RSDW, and RRDW were 78.9, 82.9, 91.8, and 71.9%, respectively, in the DC1 population; 76.4, 82.6, 73.9, and 63.8%, respectively, in the DC2 population; and 63.8, 91.1, 54.5, and 72.4%, respectively, in the 8way population. For Al, the average trait values were 90.0, 73.0, 60.8, and 50.3% for RSL, RRL, RSDW, and RRDW, respectively, in the DC1 population; 89.3, 81.3, 66.0, and 54.0%, respectively, in the DC2 population; and 88.6, 84.1, 75.2, and 61.1%, respectively, in the 8way population. Transgressive segregations were observed for SL, RL, SDW, RDW, RSL, RRL, RSDW, and RRDW of Fe, Zn and Al stresses in all MAGIC populations.

**Table 2:** Performance of nine growth traits of the parental lines and MAGIC populations under Fe, Zn, and Al stress conditions.

**Stress**	**Trait**	**MAGIC Parents**								**MAGIC Populations**		
		**A**	**B**	**C**	**D**	**E**	**F**	**G**	**H**	**DC1**	**DC2**	**8way**
		**Mean**	**Mean**	**Mean**	**Mean**	**Mean**	**Mean**	**Mean**	**Mean**	**Mean ± SD**	**Mean ± SD**	**Mean ± SD**
Fe	MTS	1.0	2.0	3.5	3.5	4.0	4.0	4.5	5.0	2.9 ± 1.3	3.0 ± 1.6	2.9 ± 1.8
	SL (cm)	36.1	41.5	39.4	37.2	34.3	40.0	37	36.4	38.6 ± 5.3	38.3 ± 4.7	42.4 ± 6.9
	RL (cm)	8.7	13.0	7.6	9.3	16.0	10.8	9.4	10.1	8.9 ± 2.1	10.1 ± 2.7	12.2 ± 2.7
	SDW (g)	0.1207	0.1433	0.1005	0.0933	0.0964	0.1246	0.0917	0.0990	0.1152 ± 0.0292	0.1208 ± 0.0300	0.1397 ± 0.0527
	RDW (g)	0.0241	0.0303	0.0233	0.0201	0.018	0.0278	0.0216	0.0247	0.0240 ± 0.0069	0.0272 ± 0.0088	0.0256 ± 0.0101
	RSL (%)	78.9	80.8	80.1	77.5	82.9	74.1	72.0	79.1	77.8 ± 9.9	77.8 ± 11.0	82.5 ± 12.0
	RRL (%)	76.5	78.8	57.4	93.6	110.3	66.4	63.7	72.9	70.2 ± 20.1	68.7 ± 18.6	114.7 ± 33.9
	RSDW (%)	67.6	77.0	75.3	82.0	111.1	69.4	70.0	82.3	82.4 ± 23.1	70.2 ± 24.3	88.4 ± 27.2
	RRDW (%)	81.9	93.2	126.4	108.0	116.0	97.6	107.8	130.5	100.9 ± 31.9	95.2 ± 39.0	113.7 ± 39.0
Zn	MTS	3.5	6.5	3.0	5.5	3.5	3.5	3.0	5.5	4.7 ± 1.8	5.5 ± 1.4	4.0 ± 1.7
	SL (cm)	37.0	35.0	39.8	34.6	36.6	41.2	39.6	33.9	39.1 ± 8.4	37.6 ± 4.8	32.6 ± 4.2
	RL (cm)	12.9	14.8	9.6	10.7	20.7	12.7	12.5	10.9	10.5 ± 2.5	12.1 ± 2.6	14.3 ± 2.4
	SDW (g)	0.1278	0.1153	0.1077	0.0919	0.1000	0.1484	0.1238	0.1047	0.1288 ± 0.0322	0.1267 ± 0.0324	0.0820 ± 0.0241
	RDW (g)	0.0162	0.0156	0.0163	0.0109	0.0160	0.0190	0.0146	0.0159	0.0168 ± 0.0047	0.0179 ± 0.0053	0.0155 ± 0.005
	RSL (%)	80.7	68.2	80.9	72.1	88.3	76.3	77.2	73.8	78.9 ± 17.5	76.4 ± 11.5	63.8 ± 9.1
	RRL (%)	113.8	89.6	72.5	107.6	142.7	78.1	84.8	79.0	82.9 ± 23.4	82.6 ± 19.2	91.1 ± 28.6
	RSDW (%)	71.6	62.0	80.8	80.7	115.3	82.7	94.5	87.1	91.8 ± 24.9	73.9 ± 26.0	54.5 ± 18.6
	RRDW (%)	55.0	48.1	88.2	58.6	103.5	66.8	73.0	83.9	71.9 ± 25.3	63.8 ± 26.1	72.4 ± 26.9
Al	MTS	–	–	–	–	–	–	–	–	–	–	–
	SL (cm)	24.3	26.8	29.1	20.3	21.2	30.4	26.7	25.5	20.3 ± 3.5	20.6 ± 3.2	25.6 ± 4.8
	RL (cm)	7.6	8.7	7.1	6.6	6.8	7.2	7.1	7.3	7.7 ± 1.4	9.5 ± 2.0	7.4 ± 1.7
	SDW (g)	0.0225	0.0138	0.0176	0.0321	0.0254	0.0332	0.0261	0.0244	0.0159 ± 0.0040	0.0181 ± 0.0049	0.0261 ± 0.0073
	RDW (g)	0.0033	0.0066	0.0046	0.0068	0.0033	0.0066	0.0055	0.0052	0.0050 ± 0.0015	0.0054 ± 0.0014	0.0055 ± 0.0021
	RSL (%)	92.4	82.4	97.8	68.9	73.4	71.0	90.4	82.3	90.0 ± 11.5	89.3 ± 12.1	88.6 ± 12.6
	RRL (%)	94.8	63.8	81.0	80.4	53.6	57.7	72.0	71.9	73.0 ± 12.9	81.3 ± 14.3	84.1 ± 19.5
	RSDW (%)	74.2	36.8	52.4	85.5	63.3	60.2	75.3	63.9	60.8 ± 13.6	66.0 ± 15.4	75.2 ± 19.6
	RRDW (%)	40.9	65.2	51.5	68.5	31.0	55.0	64.8	53.8	50.3 ± 11.7	54.0 ± 11.7	61.1 ± 20.5

### Trait correlations

**Figure 2:**
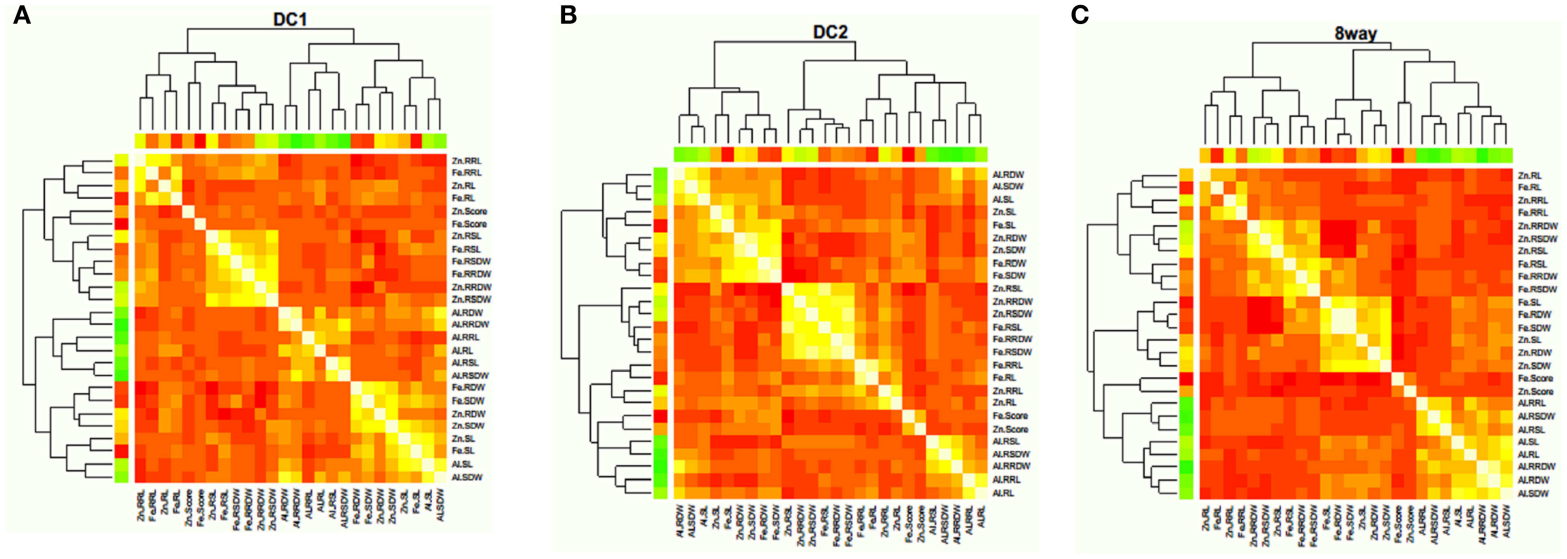
Correlation coefficients among different stress traits estimated in DC1 **(A)**, DC2 **(B)**, and 8way **(C)**, respectively. The colors showed the absolute value of corresponding r. Light yellow and red indicated high and low correlations, respectively.

The trait correlations were in the same direction for all MAGIC populations with respect to all the trait pairs (Figure 2; Supplementary Table S2). The most significant and positive correlations were observed between SL and SDW (0.741 in DC2), RL and RRL (0.672 in DC2), RDW and RRDW (0.864 in 8way), and RSL and RSDW (0.748 in DC2) for Al stress conditions. For Zn stress, these were between RL and RRL (0.694 in DC2), SDW and RDW (0.805 in DC2), and RSDW and RRDW (0.769 in DC2). For Fe stress these were between SDW and RDW (0.896 in 8way) and RL and RRL (0.817 in DC2). This was considered to be the result of the strong positive correlation observed between the different traits of SL and SDW, RL and RDW, SDW and RDW. The same trait correlations were highly significant and positive for MTS (0.335 in DC2), SL (0.761 in DC1), RSL (0.779 in DC2), and RSDW (0.795 in DC2) under Fe and Zn stress. These results strongly suggest that great potential exists for the selection of Fe and Zn toxicity-tolerant materials.

### QTL for Fe toxicity tolerance

In the five MAGIC populations, a total of 21 QTL were mapped for MTS, SL, RL, SDW, RDW, RSL, and RRDW on all the chromosomes, with the exception of chromosome 10. Among these, two, one, eight, two, and 11 were detected in the DC1, DC2, 8way, DC12, and RMPRIL populations, respectively (Table 3; Supplementary Figure S1). For MTS, three putative QTL were detected on chromosome 2. *qFeMTS2.1* and *qFeScore2.2* were identified in RMPRIL, and explained 2.2 and 2.4% of the phenotypic variation, respectively. *qFeMTS2.3* was detected in the 8way population and explained 3.9% of the phenotypic variation. For SL, seven putative QTL were detected on chromosomes 1, 3, 7, and 9. *qFeSL1.1, qFeSL3.3, qFeSL7*, and *qFeSL9* were detected in the RMPRIL population and accounted for 1.8–2.0% of the phenotypic variation, whereas *qFeSL3.1* and *qFeSL3.2* were detected in the 8way population and explained 3.5 and 4.0% of the phenotypic variation, respectively. *qFeSL1.2* was detected in the DC1, 8way, DC12, and RMPRIL populations and explained 3.6–18.9% of the phenotypic variation. For RL, three QTL were detected on chromosomes 6, 7, and 8. *qFeRL6* and *qFeRL8* were detected in the 8way population and explained 3.4 and 4.3% of the phenotypic variation. *qFeRL7* was detected in DC12 and explained 3.6% of the phenotypic variation. For SDW, two QTL were detected on chromosomes 2 and 5. *qFeSDW2* was detected in the DC1 population and accounted for 7.5% of the phenotypic variation. *qFeSDW5* was detected in the RMPRIL population and explained 1.9% of the phenotypic variation. For RDW, only one QTL, *qFeRDW5*, located on chromosome 5 was detected in the 8way population for RDW and explained 4.4% of the phenotypic variation. For RSL, four QTL were detected on chromosomes 6, 8, 11, and 12. *qFeRSL6* and *qFeRSL11* were detected in RMPRIL and explained 2.7 and 1.8% of the phenotypic variation, respectively. *qFeRSL8* and *qFeRSL12*, which explained 3.8 and 8.1% of the phenotypic variation, were detected in both the 8way and DC2 populations. For RRDW, only one QTL located on chromosome 4 was detected in the RMPRIL population, which accounted for 1.9% of the phenotypic variation. One major QTL, *qFeSL1.2*, located at 38.4 Mb on chromosome 1 was detected for SL in all populations except in DC2. The QTL explained phenotypic variation of about 18.9% in the DC1 population and 12.8% in the DC12 population. The allele from parental line C increased SL with an average effect of 6.4 and 7.6 cm.

**Table 3:** Putative QTL detected for Fe tolerance in five MAGIC populations.

**QTL**	**Alleles**	**Position**	**DC1**		**DC2**		**8way**		**DC12**		**RMPRIL**		**Gene Symbol**	**References**
			***R*^2^ (%)^a^**	**Effect^b^**	***R*^2^ (%)^a^**	**Effect^b^**	***R*^2^ (%)^a^**	**Effect^b^**	***R*^2^ (%)^a^**	**Effect^b^**	***R*^2^ (%)^a^**	**Effect^b^**		
*qFeMTS2.1*	C/T	2767000	–	–	–	–	–	–	–	–	2.2	0.7	–	–
*qFeMTS2.2*	C/T	18805689	–	–	–	–	–	–	–	–	2.4	−1.1	–	–
*qFeMTS2.3*	T/C	24494645	–	–	–	–	3.9	−1.5	–	–	–	–	–	–
*qFeSL1.1*	A/G	36300306	–	–	–	–	–	–	–	–	2	−5	MTS	Wan et al., 2005
*qFeSL1.2*	T/G	38398579	18.9	−6.4	–	–	3.6	−5.5	12.8	−7.6	6	−7.4	–	–
*qFeSL3.1*	T/C	16631905	–	–	–	–	3.5	3.6	–	–	–	–	*OsIRO3*	Zheng et al., 2010
*qFeSL3.1*	T/G	21547958	–	–	–	–	4	3.7	–	–	–	–	MTS	Wan et al., 2005
*qFeSL3.2*	A/G	36294709	–	–	–	–	–	–	–	–	1.8	3	–	–
*qFeSL7*	C/T	14615956	–	–	–	–	–	–	–	–	2	−3.7	–	–
*qFeSL9*	C/T	14162048	–	–	–	–	–	–	–	–	1.8	−2.2	–	–
*qFeRL6*	C/A	4870073	–	–	–	–	3.4	1.2	–	–	–	–	–	–
*qFeRL7*	G/A	2678714	–	–	–	–	–	–	3.6	1.4	–	–	–	–
*qFeRL8*	A/G	662509	–	–	–	–	4.3	−1.5	–	–	–	–	*IDEF1*	Kobayashi et al., 2007, 2009
*qFeSDW2*	A/C	19190121	7.5	0.045	–	–	–	–	–	–	–	–	SDW	Shimizu et al., 2005
*qFeSDW5*	A/G	14523018	–	–	–	–	–	–	–	–	1.9	−0.0283	MTS	Dufey et al., 2015
*qFeRDW5*	T/C	16622552	–	–	–	–	4.4	−0.0082	–	–	–	–	RDW	Dufey et al., 2015
*qFeRSL11*	A/C	20860944	–	–	–	–	–	–	–	–	1.8	−3.4	–	–
*qFeRSL6*	A/G	5054974	–	–	–	–	–	–	–	–	2.7	−16.7	–	–
*qFeRSL8*	A/G	2176865	–	–	–	–	3.8	−24.7	–	–	–	–	–	–
*qFeRSL12*	T/C	18022342	–	–	8.1	12.4	–	–	–	–	–	–	–	–
*qFeRRDW4*	G/A	1166749	–	–	–	–	–	–	–	–	1.9	−25.4	RRDW	Shimizu et al., 2005

a *R^2^ (%): Phenotypic variance explained*.

b *Effect: Allele effect with respect to the minor allele*.

### QTL for Zn toxicity tolerance

In the five MAGIC populations, a total of 30 QTL were mapped for nine traits on all chromosomes except 5 and 9. No SNP markers were detected to be significantly associated in the DC2 population, while six, two, eight, and 23 QTL were detected in the DC1, 8way, DC12, and RMPRIL populations, respectively (Table 4; Supplementary Figure S2). For MTS, one QTL, *qZnMTS7*, was detected on chromosome 7 and explained 7.7, 4.2, and 2.0% of the phenotypic variation in the DC1, DC12, and RMPRIL populations, respectively. For SL, 12 putative QTL were detected on chromosomes 1, 2, 3, 4, 6, 8, and 12, of which six QTL (*qZnSL1.1, qZnSL2.1, qZnSL3.1, qZnSL3.2, qZnSL8.1*, and *qZnSL8.2*) were detected in RMPRIL and explained 1.8–2.8% of the phenotypic variation. Three QTL (*qZnSL4.1, qZnSL4.2*, and *qZnSL12*) were detected in DC12 and explained 3.9–5.1% of the phenotypic variation. *qZnSL6* was detected in DC1, explaining 8.4% of the phenotypic variation, while *qZnSL2.2* was detected on chromosome 2 and explained 2.7–8.8% of the phenotypic variation in DC1, DC12, and RMPRIL, respectively. *qZnSL1.2* was associated in the DC1, 8way, DC12, and RMPRIL populations, and explained 6.2–11.8% of the phenotypic variation. For RL, four QTL were detected on chromosomes 2, 6, and 11, the *qZnRL2* was detected in the DC1 population and explained 9.0% of the phenotypic variation. *qZnRL6.1, qZnRL6.2*, and *qZnRL11* were detected in the RMPRIL population and explained 1.7–3.7% of the phenotypic variation. For SDW, five QTL were detected on chromosomes 1, 2, 3, 4, and 8. *qZnSDW1, qZnSDW2, qZnSDW3, qZnSDW4*, and *qZnSDW8* were detected in the RMPRIL population and explained 3.6% of the phenotypic variation. For RDW, two QTL *qZnRDW2.1* and *qZnRDW2.2* were detected on chromosome 2 in the DC12 and RMPRIL populations, respectively, accounting for 3.7 and 2.3% of the phenotypic variation. For RSL, two QTL were detected on chromosomes 2 and 4. *qZnRSL2* was detected in both the DC1 and RMPRIL populations and explained 7.8 and 2.4% of the phenotypic variation, respectively. *qZnRSL4* was detected in DC12 and RMPRIL and was associated with 3.7 and 1.8% of the phenotypic variation, respectively. For RSDW, two QTL (*qZnRSDW8* and *qZnRSDW10*) were detected on chromosomes 8 and 10 in the 8way and RMPRIL populations, explaining 4.1 and 1.9% of the phenotypic variation, respectively. For RRL, a single QTL, *qZnRRL3*, was detected in RMPRIL and explained 1.8% of the phenotypic variation. For RRDW, one QTL *qZnRRDW4* was detected on chromosome 4 in the RMPRIL population, which explained 2.1% of the phenotypic variation. One major QTL *qZnSL1.2* (chr.1: 38.4 Mb) was detected for SL in all populations except DC2, which explained 11.8 and 11.3% of the phenotypic variation in DC1 and DC12, respectively. The allele from parental line C increased SL with an average effect of 6.7 and 9.1 cm.

**Table 4:** Putative QTL detected for Zn tolerance in five MAGIC populations.

**QTL**	**Alleles**	**Position**	**DC1**		**DC2**		**8way**		**DC12**		**RMPRIL**		**Gene Symbol**	**References**
			***R*^2^ (%)^a^**	**Effect^b^**	***R*^2^ (%)^a^**	**Effect^b^**	***R*^2^ (%)^a^**	**Effect^b^**	***R*^2^ (%)^a^**	**Effect^b^**	***R*^2^ (%)^a^**	**Effect^b^**		
*qZnScore7*	G/A	4498977	7.7	−1.8	–	–	–	–	4.2	−1.7	2	−1.4	–	–
*qZnSL1.1*	T/G	35505539	–	–	–	–	–	–	–	–	2.8	−3.8	–	–
*qZnSL1.2*	T/G	38398579	11.8	−6.7	–	–	6.2	−4.2	11.3	−9.1	7.2	−6.3	–	–
*qZnSL2.1*	G/T	2387479	–	–	–	–	–	–	–	–	1.8	−2.6	–	–
*qZnSL2.2*	C/T	30673065	8.8	−11.2	–	–	–	–	7.5	−11.4	2.7	−7.1	–	–
*qZnSL3.1*	C/T	6950989	–	–	–	–	–	–	–	–	1.9	2.3	*OsFRDL1*	Yokosho et al., 2009
*qZnSL3.2*	A/G	36152527	–	–	–	–	–	–	–	–	2	2.3	–	–
*qZnSL6*	T/C	16861539	8.4	3.3	–	–	–	–	–	–	–	–	–	–
*qZnSL4.1*	A/G	25681135	–	–	–	–	–	–	4	−4.9	–	–	SDW	Zhang et al., 2013
*qZnSL4.2*	A/G	30976306	–	–	–	–	–	–	5.1	3.9	–	–	*OsZIP3*	Sasaki et al., 2015
*qZnSL8.1*	G/A	2421740	–	–	–	–	–	–	–	–	1.9	2.9	–	–
*qZnSL8.2*	C/T	5137795	–	–	–	–	–	–	–	–	2.3	−1.9	*OsZIP4*	Ishimaru et al., 2005
*qZnSL12*	T/C	9996292	–	–	–	–	–	–	3.9	−4.6	–	–	–	–
*qZnRL2*	C/T	23207588	9	−1.7	–	–	–	–	–	–	–	–	–	–
*qZnRL6.1*	A/G	2945886	–	–	–	–	–	–	–	–	1.7	−1	–	–
*qZnRL6.2*	T/C	30619612	–	–	–	–	–	–	–	–	1.8	1.4	*OsHMA2*	Takahashi et al., 2012
*qZnRL11*	G/A	9235660	–	–	–	–	–	–	–	–	1.8	−1	–	–
*qZnSDW1*	A/C	38502586	–	–	–	–	–	–	–	–	2	−0.0189	–	–
*qZnSDW2*	T/C	25490698	–	–	–	–	–	–	–	–	1.7	0.0155	–	–
*qZnSDW3*	G/A	1235607	–	–	–	–	–	–	–	–	3.7	0.0168	–	–
*qZnSDW4*	C/T	33294120	–	–	–	–	–	–	–	–	1.8	0.0127	–	–
*qZnSDW8*	G/A	2421740	–	–	–	–	–	–	–	–	1.9	0.017	–	–
*qZnRDW2.1*	T/C	10787293	–	–	–	–	–	–	3.7	0.0027	–	–	RDW	Zhang et al., 2013
*qZnRDW2.2*	T/C	25835210	–	–	–	–	–	–	–	–	2.3	−0.0022	SDW	Zhang et al., 2013
*qZnRSL2*	T/C	30496195	7.8	−40	–	–	–	–	–	–	2.4	−16.7	–	–
*qZnRSL4*	C/A	27676200	–	–	–	–	–	–	3.7	−12.5	1.8	−7.8	–	–
*qZnRSDW8*	A/T	886967	–	–	–	–	4.1	−10.7	–	–	–	–	–	–
*qZnRSDW10*	A/G	14980285	–	–	–	–	–	–	–	–	1.9	−11.3	–	–
*qZnRRL3*	A/G	12548747	–	–	–	–	–	–	–	–	1.8	13.6	–	–
*qZnRRDW4*	G/A	1166749	–	–	–	–	–	–	–	–	2.1	−16.8	–	–

a *R^2^ (%): Phenotypic variance explained*.

b *Effect: Allele effect with respect to the minor allele*.

### QTL for Al toxicity tolerance

A total of 21 QTL were mapped for the eight traits on all chromosomes except chromosome 4. Among them, seven, three, three, five, and 10 QTL were detected in the DC1, DC2, 8way, DC12, and RMPRIL populations, respectively (Table 5; Supplementary Figure S3). For SL, three putative QTL were detected on chromosomes 1 and 3. *qAlSL1.1* was detected in DC2 and explained 4.0% of the phenotypic variation, while *qAlSL3* was detected in the RMPRIL population and explained 2.8% of the phenotypic variation. *qAlSL1.2* was detected in the DC1, 8way, DC12, and RMPRIL populations and explained 6.2–15.0% of the phenotypic variation. For RL, seven QTL were detected on chromosomes 1, 6, and 12. *qAlRL1.1* and *qAlRL1.2* were detected in the DC2 population and both explained 0.1% of the phenotypic variation. *qAlRL6, qAlRL12.1*, and *qAlRL12.2* were detected in RMPRIL, explaining 1.9, 1.9, and 2.1% of the phenotypic variation, respectively. *qAlRL1.3* was detected in the DC1 and DC12 populations and explained 9.4 and 5.1% of the phenotypic variation, respectively. *qAlRL1.4* was detected in the DC2 and RMPRIL populations and accounted for 0.1 and 2.2% of the phenotypic variation, respectively. For SDW, five QTL were detected on chromosomes 1, 5, 6, 7, and 9. *qAlSDW1* was detected in 8way and explained 4.2% of the phenotypic variation. *qAlSDW6* was detected in the DC1 population and explained 9.2% of the phenotypic variation. *qAlSDW7* and *qAlSDW9* were detected in the RMPRIL population and accounted for 2.0 and 2.2% of the phenotypic variation, respectively. *qAlSDW5* was detected in the DC1 and DC12 populations and explained 10.2 and 4.9% of the phenotypic variation, respectively. For RDW, *qAlRDW3* was detected in the DC1 and DC12 populations and explained 8.2 and 4.4% of the phenotypic variation, respectively. *qAlRDW6* was detected in the 8way population and explained 4.4% of the phenotypic variation. For RSL, RRL, RSDW, and RRDW, only one QTL was detected in the single population. *qAlRSL11, qAlRRL2, qAlRSDW10*, and *qAlRRDW12* were detected in the 8way, RMPRIL, DC1, and RMPRIL populations on chromosomes 11, 2, 10, and 12 and explained 2.0–9.6% of the phenotypic variation. Two major QTL were detected for SL and SDW. The major QTL *qAlSL1.2* (chr.1: 38.4 Mb) was detected in all populations except in DC2, with a phenotypic variation of 15.0% for the DC1 population and 14.6% for the 8way population. The allele from parental line C increased SL with an average effect of 3.7 and 8.0 cm. Another major QTL *qAlSDW5* (chr.5: 5.4 Mb) was detected in the DC1 and DC12 populations and explained 10.2 and 4.9% of the phenotypic variation, respectively. The allele from parental line A increased SDW with an average effect of 0.0037 g.

**Table 5:** Putative QTL detected for Al tolerance in five MAGIC populations.

**QTL**	**Alleles**	**Position**	**DC1**		**DC2**		**8way**		**DC12**		**RMPRIL**		**Gene Symbol**	**References**
			***R*^2^ (%)^a^**	**Effect^b^**	***R*^2^ (%)^a^**	**Effect^b^**	***R*^2^ (%)^a^**	**Effect^b^**	***R*^2^ (%)^a^**	**Effect^b^**	***R*^2^ (%)^a^**	**Effect^b^**		
*qAlSL1.1*	C/T	5969153	–	–	–	–	–	–	4.0	−1.8	–	–	–	–
*qAlSL1.2*	T/C	38381991	15	−3.7	–	–	14.6	−8	9.9	−4.3	6.2	−5.7	*OsMGT1*	Chen et al., 2012
*qAlSL3*	T/C	35466303	–	–	–	–	–	–	–	–	2	−1.8	*Os03g0760800*	Yamaji et al., 2009
*qAlRL1.1*	G/A	9272759	–	–	0.1	−3.7	–	–	–	–	–	–	–	–
*qAlRL1.2*	G/A	18735343	–	–	0.1	−4.2	–	–	–	–	–	–	–	–
*qAlRL1.3*	G/A	35465639	9.4	1.2	–	–	–	–	5.1	1.2	–	–	*Alt_*TRG*_ 1.1*	Famoso et al., 2011
*qAlRL1.4*	C/A	38207031	–	–	0.1	1.7	–	–	–	–	2.2	1.0	*OsMGT1*	Chen et al., 2012
*qAlRL6*	A/G	1968315	–	–	–	–	–	–	–	–	1.9	−0.6	–	–
*qAlRL12.1*	C/T	17406215	–	–	–	–	–	–	–	–	1.9	−0.6	–	–
*qAlRL12.2*	C/T	23253056	–	–	–	–	–	–	–	–	2.1	−0.6	*Alt_*TRG*_ 12.2*	Famoso et al., 2011
*qAlSDW1*	C/T	38381358	–	–	–	–	4.2	−0.0064	–	–	–	–	*OsMGT1*	Chen et al., 2012
*qAlSDW5*	T/C	5368362	10.2	−0.0037	–	–	–	–	4.9	−0.0037	–	–	*LOC_Os05g09440*	Krill et al., 2010
*qAlSDW6*	A/G	21082410	9.2	−0.0026	–	–	–	–	–	–	–	–	*LOC_Os06g36450*	Krill et al., 2010
*qAlSDW7*	G/A	8231321	–	–	–	–	–	–	–	–	2	−0.0031	–	–
*qAlSDW9*	C/A	14688117	–	–	–	–	–	–	–	–	2.2	0.0022	*qRRE-9*	Xue et al., 2006a
*qAlRDW3*	T/C	12767754	8.2	0.0012	–	–	–	–	4.4	0.0011	–	–	*LOC_Os03g21950*	Krill et al., 2010
*qAlRDW6*	G/T	19697887	8.1	0.0011	–	–	–	–	–	–	–	–	–	–
*qAlRSL11*	C/T	5880923	–	–	–	–	4.4	14.2	–	–	–	–	*qRRE-11*	Xue et al., 2006b
*qAlRRL2*	T/C	31634550	–	–	–	–	–	–	–	–	2.0	−7.0	*LOC_Os02g51930*	Yamaji et al., 2009
*qAlRSDW10*	T/C	16169758	9.6	−31.7	–	–	–	–	–	–	–	–	–	–
*qAlRRDW12*	T/C	22035605	–	–	–	–	–	–	–	–	2.0	6.6	–	–

a *R^2^ (%): Phenotypic variance explained*.

b *Effect: Allele effect with respect to the minor allele*.

### QTL clusters

**Figure 3:**
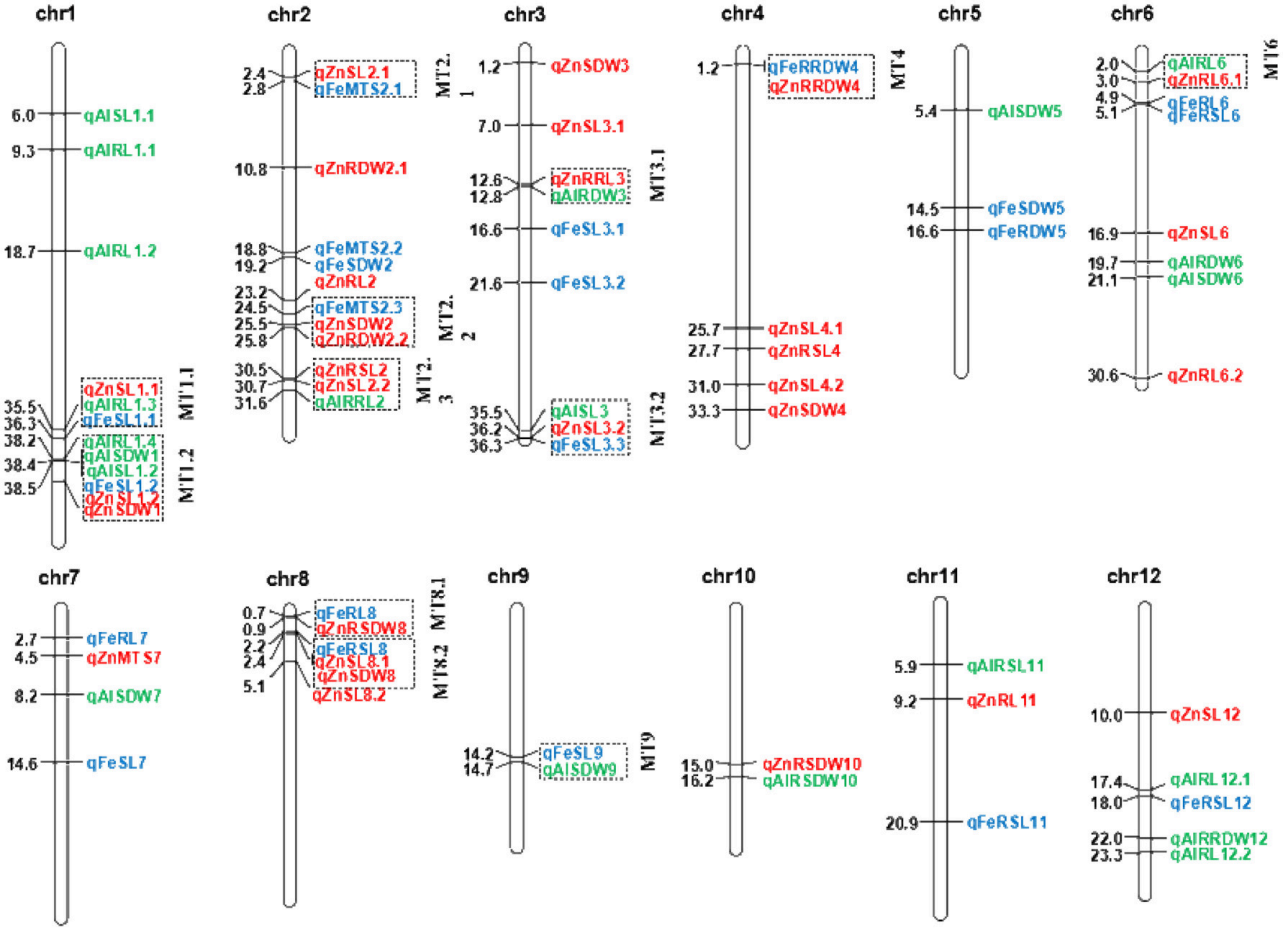
Positions of the QTL for SL, RL, SDW, RDW, RSL, RRL, RSDW, RRDW, and MTS on all chromosomes under Fe, Zn, and Al stress conditions. Blue, red, and green correspond to the Fe, Zn, and Al QTL detected, respectively. Individual QTL are designated with the italicized abbreviation of the trait and the chromosome number. When more than one QTL affecting a trait is identified on the same chromosome, they are distinguished by decimal numbers. The distances between the markers (Mb) are listed to the left of each figure. The clustered QTL for different tolerance traits are indicated using dashed outline boxes.

A total of 33 QTL were grouped into 12 clusters on chromosomes 1, 2, 3, 4, 6, 8, and 9 (Figure 3). Three QTL clusters were detected under three stress conditions. The cluster MT1.1 at 35.4-36.3 Mb was covered by *qAlRL1.3, qZnSL1.1*, and *qFeSL1.1*, while MT1.2 at 38.2–38.5 Mb was covered by *qFeSL1.2, qZnSL1.2, qZnSDW1, qAlRL1.4, qAlSDW1*, and *qAlSL1.2*. Cluster MT3.2 at 35.4–36.2 Mb was covered by *qAlSL3, qZnSL3.2*, and *qFeSL3.3*. Five QTL clusters, MT2.1 (*qZnSL2.1* and *qFeMTS2* at 2.4–2.8 Mb), MT2.2 (*qFeMTS2, qZnSDW2*, and *qZnRDW2.2* at 24.5–25.8 Mb), MT4 (*qFeRRDW4* and *qZnRRDW4* at 1.2 Mb), MT8.1 (*qFeRL8* and *qZnRSDW8* at 0.7–0.9 Mb), and MT8.2 (*qFeRSL8, qZnSL8.1*, and *qZnSDW8* at 2.2–2.4 Mb), were detected under both Fe and Zn stress conditions. Three QTL clusters, MT2.3 (*qZnRSL2, qZnSL2.2*, and *qAlRRL2* at 30.5–31.6 Mb), MT3.1 (*qZnRRL3* and *qAlRDW3* at 12.5–12.8 Mb), and MT6 (*qAlRL6* and *qZnRL6.1* at 2.0–3.0 Mb) were detected under both Zn and Al stress conditions. The QTL cluster MT9.1 (*qFeSL9* and *qAlSDW9* at 14.2–14.7 Mb) was detected under the Fe and Al treatments. The results suggest that these chromosomal regions are significantly associated with metal tolerance and might be pleiotropic or encode multiple tightly linked genes.

## Discussion

### Mapping power and resolution of the magic populations

We were able to overcome the limitations of bi-parental populations, in which only two alleles are analyzed and the genetic recombination is limited. In this study by using multiple parents to produce mapping populations with high allelic and phenotypic diversity, in combination with high levels of recombination events brought about by several cycles of intermating. The efficacy of MAGIC populations for high resolution mapping in rice has been previously reported (Bandillo et al., 2013; Li et al., 2013; Meng et al., 2016a,b). The eight parents used for the MAGIC population in this study displayed obvious differences in agronomic traits and biotic/abiotic stresses, such as resistance to drought, salinity, ferrous, zinc, aluminum, cadmium, and bacterial blight (Table 1). These populations possess a slightly higher genetic diversity than a population of 248 of IRRI's breeding lines (Meng et al., 2016a). Due to the multiple hybridizations and selfing used in developing the populations, the DC1, DC2, and 8way populations exhibited no clear population structure and thus the effect of population structure on the mapping results is negligible. These populations were also used to identify QTL for 14 yield-related traits in the dry season (DS) and wet season (WS) during 2014 at the headquarters of the IRRI (Meng et al., 2016b). The mapping resolution and power of the DC12 and RMPRIL populations was higher than the DC1, DC2, and 8way populations as a result of the larger population size of the former. Greater metal toxicity-tolerant QTL were identified in the RMPRIL population (44 QTL) in comparison to the DC12 (15 QTL), 8way (13 QTL), DC1 (15 QTL), and DC2 (four QTL) populations. A total of 11 QTL were detected across the different MAGIC populations, particularly *qFeSL1.2, qZnSL1.2*, and *qAlSL1.2*, which were identified in all the populations except DC2. The QTL corresponding to known genes were also identified in the RMPRIL (13 QTL), DC12 (7 QTL), 8way (seven QTL), DC1 (six QTL), and DC2 (one QTL) populations. This suggests that the combined populations possess substantially higher QTL detection power and precision brought by the large population size and high level of recombination (Coles et al., 2010; Steinhoff et al., 2011). Although the MAGIC populations exhibited no clear population structure, approximately 20 lines showed exceptionally high kinship, and 32 lines were slightly different compared to the other lines in the DC2 population (Meng et al., 2016a). These results explain the observed low detection power in DC2 compared with the DC1 and 8way populations. The use of DC12 (DC1 + DC2) and RMPRIL (DC1 + DC2 + 8way) improved the mapping power and resolution. In addition, the mapping resolution in previously reported studies was limited by low marker density (several hundreds), which only covered a limited number of chromosomal recombination events occurring in biparental crosses (Huang and Han, 2014). In the present study, the high density 55 K rice SNP array genotyping platform was used to increase the mapping resolution.

### Comparison of identified and reported QTL

In the present study, a total of 21, 30, and 21 putative QTL were detected under treatment with Fe, Zn, and Al, respectively. With respect to Fe tolerance, *qFeSL1* and *qFeSL3*, located on chromosomes 1 and 3, were mapped closely to the leaf bronzing score-based QTL identified by Wan et al. (2005). *qFeRDW5* and *qFeSDW5* on chromosome 5 co-located with the QTL detected by Dufey et al. (2015) for RDW and MTS. *qFeSDW2* co-localized with the QTL detected for SDW and MTS by Shimizu et al. (2005), and *qFeSL3* associated with SL (Chr.3: 16.6 Mb) detected in the 8way population was located 1 Mb away from the previously reported *OsIRO3* gene (helix-loop-helix family transcription factor) (Zheng et al., 2010). The *qFeRL8* gene associated with RL detected in 8way (Chr.8: 0.7 Mb) was located 0.5 Mb away from the *IDEF1* gene (Iron Deficiency-responsive Element-binding Factor 1) (Inoue et al., 2009).

The QTL peaks of *qZnRDW2, qZnRDW2*, and *qZnSL4* on chromosomes 2 and 5 were previously reported by Zhang et al. (2013) using reciprocal advanced backcross introgression lines (cross between Lemont and Teqing) for RDW and SDW traits. The Zn tolerance-related QTL located with the genes responsible for the translocation of metal in plants. The *qZnSL3* (Chr.8: 6.9 Mb) gene was located 0.8 Mb away from the *OsFRDL1* gene (citrate transporter), which is required for the translocation of iron (Yokosho et al., 2009). The *qZnRL6* (Chr.6: 30.6 Mb) gene was located 264 kb away from the *OsHMA2* gene (heavy metal ATPase 2), a P1B-ATPase that is involved in the root-to-shoot translocation of Zn and Cd in rice (Satoh-Nagasawa et al., 2012; Takahashi et al., 2012). The *qZnSL8* (Chr.9: 5.1 Mb) gene was located 1.1 Mb away from the *OsZIP4* gene; a zinc-regulated zinc transporter (Ishimaru et al., 2005). The *qZnSL4* (Chr.4: 30.1 Mb) gene was positioned at 102 kb away from the *OsZIP3* gene; a zinc transporter (Sasaki et al., 2015).

Twelve of the 21 QTL for Al toxicity tolerance co-localized with known genes and previously mapped QTL. Three QTL, namely *qAlSL1, qAlRL1*, and *qAlSDW1*, located at 38.2–38.4 on chromosome 1, co-localized with the known gene *OsMGT1* at 39,419,964 bp. The *OsMGT1* gene acts as an Mg transporter in the roots and its up-regulation is required for conferring Al tolerance in rice (Chen et al., 2012). The *qAlRL1.3* (Chr.1: 35.5 Mb) and *qAlRL12.2* (Chr.12: 23.3 Mb) co-localized with two QTL, *Alt*_*TRG*_ 1.1 and *Alt*_*TRG*_ 12.2, for total root growth (Dufey et al., 2015). Three QTL, *qAlRDW3* (Chr.3: 12.8 Mb), *qAlSDW5* (Chr.5: 5.4 Mb), and *qAlSDW6* (Chr.6: 21.1 Mb) co-localized with the genes *LOC_Os03g21950, LOC_Os05g09440*, and *LOC_Os06g36450*, respectively. These genes constitute rice orthologs of a maize isocitrate lyase, which is an a priori candidate gene associated with Al tolerance in maize (Krill et al., 2010). *qAlRRL2* (Chr.2: 31.6 Mb) was found to co-localize with the gene *LOC_Os02g51930*, while *qAlSL3* (Chr.3: 35.5 Mb) localized with the *Os03g0760800*; both of which were detected by microarray analysis using the *art1* mutant and wild-type rice (Yamaji et al., 2009). The *qAlSDW9* (Chr.9: 14.7 Mb) and *qAlRSL11* (Chr.11: 5.9 Mb) genes co-localized with two QTL, namely *qRRE-9* and *qRRE-11*, detected for relative root elongation via biparental linkage mapping (Xue et al., 2006a,b). The QTL *qFeSL1* (38.4 Mb, *p* = 8.9E-13 in DC12 and RMPRIL), *qZnSL1*(38.4 Mb, *p* = 9.2E-14 in 8way), and *qAlSL1* (38.4 Mb, *p* = 5.1E-15 in RMPRIL) associated with shoot length at seedling stage under three metal conditions were located in the region harboring the QTL for plant height (Lee et al., 2014), seedling shoot length (Abe et al., 2012) and seed dormancy (Ye et al., 2015) using biparental and plant height MAGIC populations (Meng et al., 2016a,b).

### Multi-trait QTL for tolerance to Fe, Zn, and Al toxicity

Thirty-three of the identified QTL in the present study occurred in 12 regions on chromosomes 1, 2, 3, 4, 6, 8, and 9, each of which harbor QTL for more than two metal conditions (Figure 3). Five chromosomal regions were repeatedly covered by overlapping QTL for Fe and Zn stress. This is believed to be caused by the strong correlation observed between Fe and Zn stress tolerance in all the populations (Supplementary Table S2). Hence, it is plausible that the QTL for Fe and Zn were identified in the same QTL cluster. In previous QTL analyses it has been observed that the QTL for significantly correlated traits are usually located in the same chromosomal region. Two Zn toxicity tolerance-related QTL, *QSdw2a* and *QSdw5*, were mapped together with the Fe toxicity tolerance QTL (Zhang et al., 2013). One Fe toxicity tolerance QTL (*qFRSDW11*) and three Zn toxicity tolerance QTL (*qZRRDW11, qZRTDW11*, and *qZRSDW11-1*) were previously mapped together (Liu et al., 2016). In studies of tetraploid and hexaploid wheat, a grain Zn QTL on chromosome 2B co-localized with a grain Fe QTL, suggesting the possibility of simultaneous improvement of Fe and Zn tolerance (Velu et al., 2017). *OsIRT1* (iron-regulated metal transporter) is not only a functional metal transporter for iron, and also involve as transporter for Zn and Cd (Lee and An, 2009). *OsYSL2* is a rice metal-NA transporter that is responsible for the phloem transport of iron and manganese, including the translocation of iron and manganese into the grain (Koike et al., 2004). Expression of a rice NAS gene, *OsNAS3*, led to increased tolerance to Fe and Zn deficiencies and to excess metal (Zn, Cu, and Ni) toxicities (Lee and An, 2009). *OZT1* confers plant tolerance to Zn and Cd ions (Lan et al., 2013). P1B-ATPases (also known as Heavy Metal ATPases: HMAs) are energized by ATP hydrolysis, and translocate heavy metals (Zn, Co, Cu, Cd, and Pb) out of cytoplasm (to plasma membrane and into vacuole) and thus play important roles in their transport, compartmentalization and detoxification. *OsHMA2* transporter is involved in root-to-shoot translocation of Zn and Cd in rice (Satoh-Nagasawa et al., 2012; Takahashi et al., 2012). *OsHMA3* can reduce the toxicity of Cd to rice seedling and maintains Zn balance in the rice stem (Sasaki et al., 2014). *OsHMA4* is a causal gene for quantitative trait locus controlling Cu accumulation in rice grain (Huang et al., 2016). Heavy metal P-type ATPase, *OsHMA5* and *OsHMA4*, involves in xylem loading of Cu in rice (Deng et al., 2013; Huang et al., 2016). In addition, more overlapping regions were detected for Fe and Zn tolerance QTL compared to previous studies (Zhang et al., 2013; Liu et al., 2016). A total of three and six multi-trait QTL were identified under three metal conditions with extremely significant values, *p* = 7.4E-7 and 5.1E-15, and were closely positioned (MT1.1 for SL and RL; MT1.2 for SL, RL, and SDW) at 35.5 Mb and 38.4 Mb of chromosome 1, respectively. The direction of the effect of all the clustered QTL detected for different tolerance traits were always equivalent, which may be caused by the pleiotropic effects of the same gene. Genetic overlaps for tolerance to different stresses have been reported in various stress conditions, including salt and drought (Wang et al., 2012) and blast resistance and drought (Xiong and Yang, 2003). These two aforementioned regions were previously reported to be associated with plant height using the same MAGIC populations (Meng et al., 2016a,b) as well as seedling shoot length (Abe et al., 2012). The 13 promising lines with MTS = 1 were selected under both Fe and Zn conditions, they are all from 8way populations. In our previous study, we also found that 8way was more powerful than the DC1 and DC2 populations for QTL identification useful for breeding (Meng et al., 2016a). The 13 ILs had an average higher RRL than average of eight parents by 29.5% (27.6%) under the Fe conditions and by 27.6% (22.3%) under Zn stress, respectively. The 13 IL_S_ could be used as resistant donors in for breeding. For *qFeMTS2.1*, all lines had the same favorable allele from parental line A, C, D, E, F and G. The results indicated that the MAGIC populations are ideal material for genetic study and marker-assisted breeding (MAB), showing a tight integration of genetic research and breeding application in rice. Genetically overlapping loci can be easily exploited in the development of cultivars with tolerance to multiple metal toxicities, as it simplifies the process of MAB and is also associated with reduced costs.

## Conclusion

The present study has identified the QTL associated with the toxicity tolerance of rice to three essential metal (Fe, Zn, and Al). As resistance cannot be measured directly, several parameters such as MTS and decreased biomass production were selected as indicators. A total of 21, 30, and 21 QTL were detected for traits related to Fe, Zn, and Al toxicity tolerance, respectively. Twenty-seven of the 72 QTL are in close proximity of previously reported QTL/genes for metal toxicity tolerance. Furthermore, three clusters MT1.1 (chr.1: 35.4–36.3 Mb), MT1.2 (chr.1: 35.4–36.3 Mb), and MT3.2 (chr.3: 35.4–36.2 Mb) were identified under all three metal conditions. The regions of the end of chromosomes 1 and 3 may play important roles in ion homeostasis and the maintenance of high biomass when rice is grown under different metal stress conditions. These results provide an opportunity to develop novel metal tolerant rice varieties via MAS.

## Author contributions

QQ and GY designed the experiment; LM, XZ, and KP performed all the phenotypic evaluation; LM, BW, and XZ performed analysis and interpretation of the data; LM and GY drafted the manuscript; all authors revised the paper and approved the final version to be published.

### Conflict of interest statement

The authors declare that the research was conducted in the absence of any commercial or financial relationships that could be construed as a potential conflict of interest. The reviewer LG declared a shared affiliation, with no collaboration, with one of the authors QQ to the handling Editor.

## Supplementary material

Supplementary Figure S1 Genome-wide association mapping for Fe tolerance in DC1 **(A)**, DC2 **(B)**, 8way **(C)**, DC12 **(D)**, and RMPRIL **(E)** populations. Manhattan plots for Fe tolerance at the seedling stage (red, khaki, blue, magenta, and green dots represent significant loci for traits RDW (RRDW), RL (RRL), SDW (RSDW), SL (RSL) and MTS in Fe stress condition, respectively) are on the left side of **(A–E)**, horizontal short dash line on the Manhattan plot indicates the genome-wide significance threshold p ≤ 0.0001; Quantile-Quantile plots for Fe tolerance are on the right side of **(A–E)**.

Supplementary Figure S2 Genome-wide association mapping for Zn tolerance in DC1 **(A)**, DC2 **(B)**, 8way **(C)**, DC12 **(D)**, and RMPRIL **(E)** populations. Manhattan plots for Zn tolerance at the seedling stage (red, khaki, blue, magenta, and green dots represent significant loci for traits RDW (RRDW), RL (RRL), SDW (RSDW), SL (RSL), and MTS in Zn stress condition, respectively) are on the left side of **(A–E)**, horizontal short dash line on the Manhattan plot indicates the genome-wide significance threshold *p* ≤ 0.0001; Quantile-Quantile plots for Zn tolerance are on the right side of **(A–E)**.

Supplementary Figure S3 Genome-wide association mapping for Al tolerance in DC1 **(A)**, DC2 **(B)**, 8way **(C)**, DC12 **(D)**, and RMPRIL **(E)** populations. Manhattan plots for Al tolerance at the seedling stage (red, khaki, blue, magenta, and green dots represent significant loci for traits RDW (RRDW), RL (RRL), SDW (RSDW), and SL (RSL) in Al stress condition, respectively) are on the left side of **(A–E)**, horizontal short dash line on the Manhattan plot indicates the genome-wide significance threshold *p* ≤ 0.0001; Quantile-Quantile plots for Al tolerance are on the right side of **(A–E)**.

Supplementary Table S1 Summary of the SNP selection procedure for the construction of the genetic map.

Supplementary Table S2 Correlation coefficients between different traits estimated under Fe, Zn, and Al conditions in the DC1, DC2, and 8way populations.
