# Supplementary figures and images for: Association Mapping of Ferrous, Zinc, and Aluminum Tolerance at the Seedling Stage in Indica Rice using MAGIC Populations
Source: Front Plant Sci. 2017 Oct 26;8:1822. doi: 10.3389/fpls.2017.01822 (PMC5662918; doi:10.3389/fpls.2017.01822)

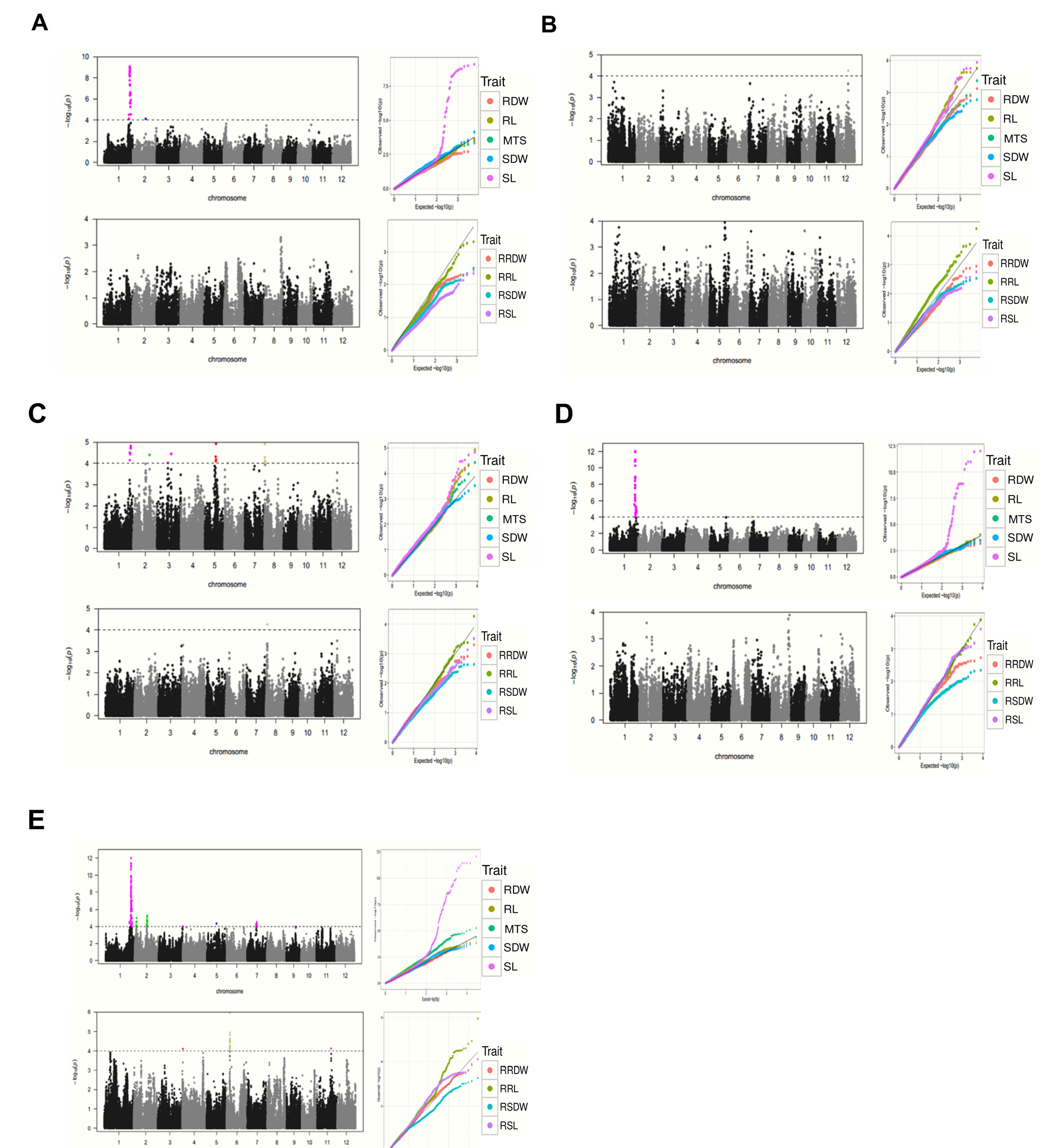

Supplement: Supplementary Figure S1 — Genome-wide association mapping for Fe tolerance in DC1 (A), DC2 (B), 8way (C), DC12 (D), and RMPRIL (E) populations. Manhattan plots for Fe tolerance at the seedling stage (red, khaki, blue, magenta, and green dots represent significant loci for traits RDW (RRDW), RL (RRL), SDW (RSDW), SL (RSL) and MTS in Fe stress condition, respectively) are on the left side of (A–E), horizontal short dash line on the Manhattan plot indicates the genome-wide significance threshold p ≤ 0.0001; Quantile-Quantile plots for Fe tolerance are on the right side of (A–E). [file Image1.TIF]

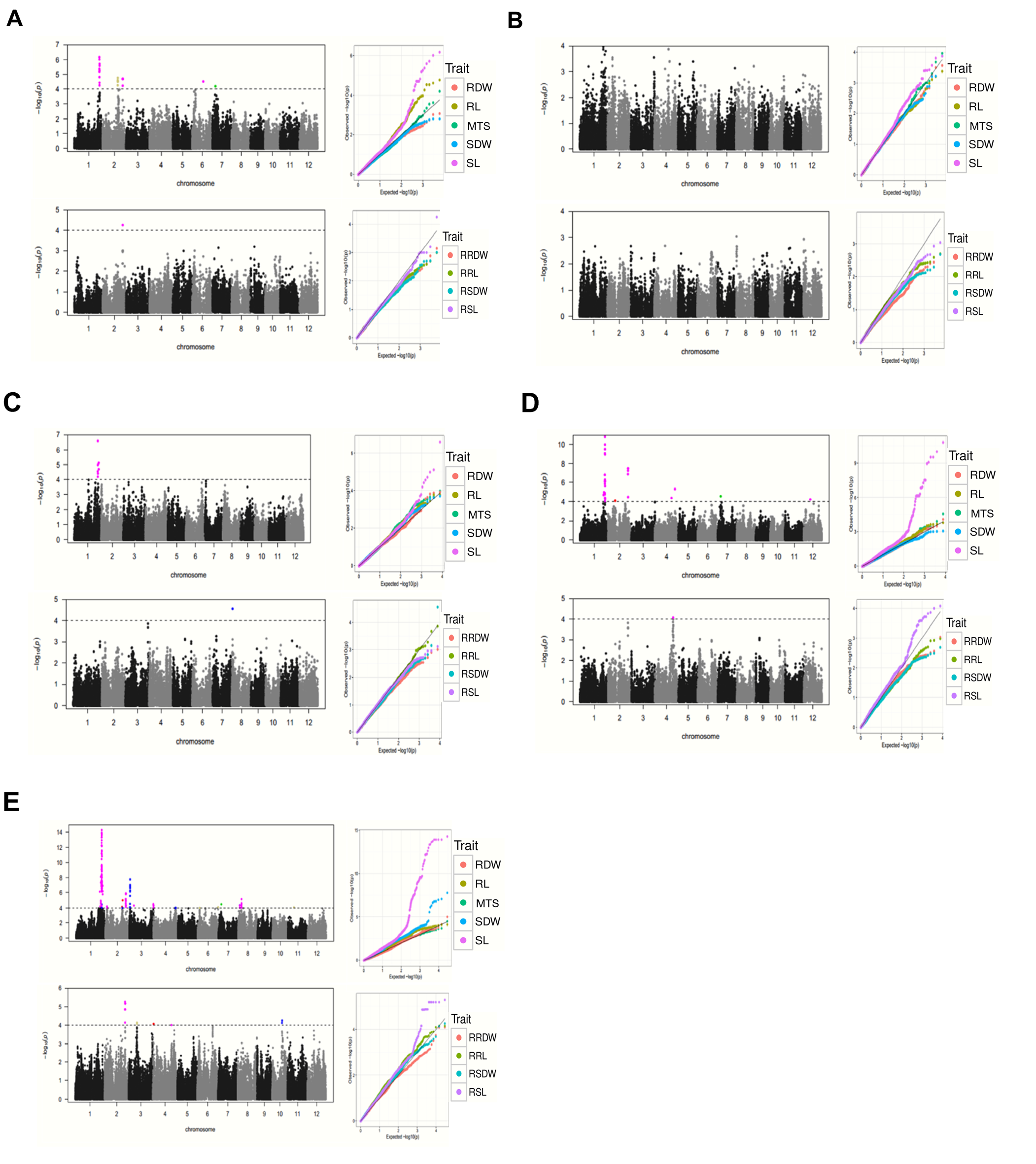

Supplement: Supplementary Figure S2 — Genome-wide association mapping for Zn tolerance in DC1 (A), DC2 (B), 8way (C), DC12 (D), and RMPRIL (E) populations. Manhattan plots for Zn tolerance at the seedling stage (red, khaki, blue, magenta, and green dots represent significant loci for traits RDW (RRDW), RL (RRL), SDW (RSDW), SL (RSL), and MTS in Zn stress condition, respectively) are on the left side of (A–E), horizontal short dash line on the Manhattan plot indicates the genome-wide significance threshold p ≤ 0.0001; Quantile-Quantile plots for Zn tolerance are on the right side of (A–E). [file Image2.TIF]

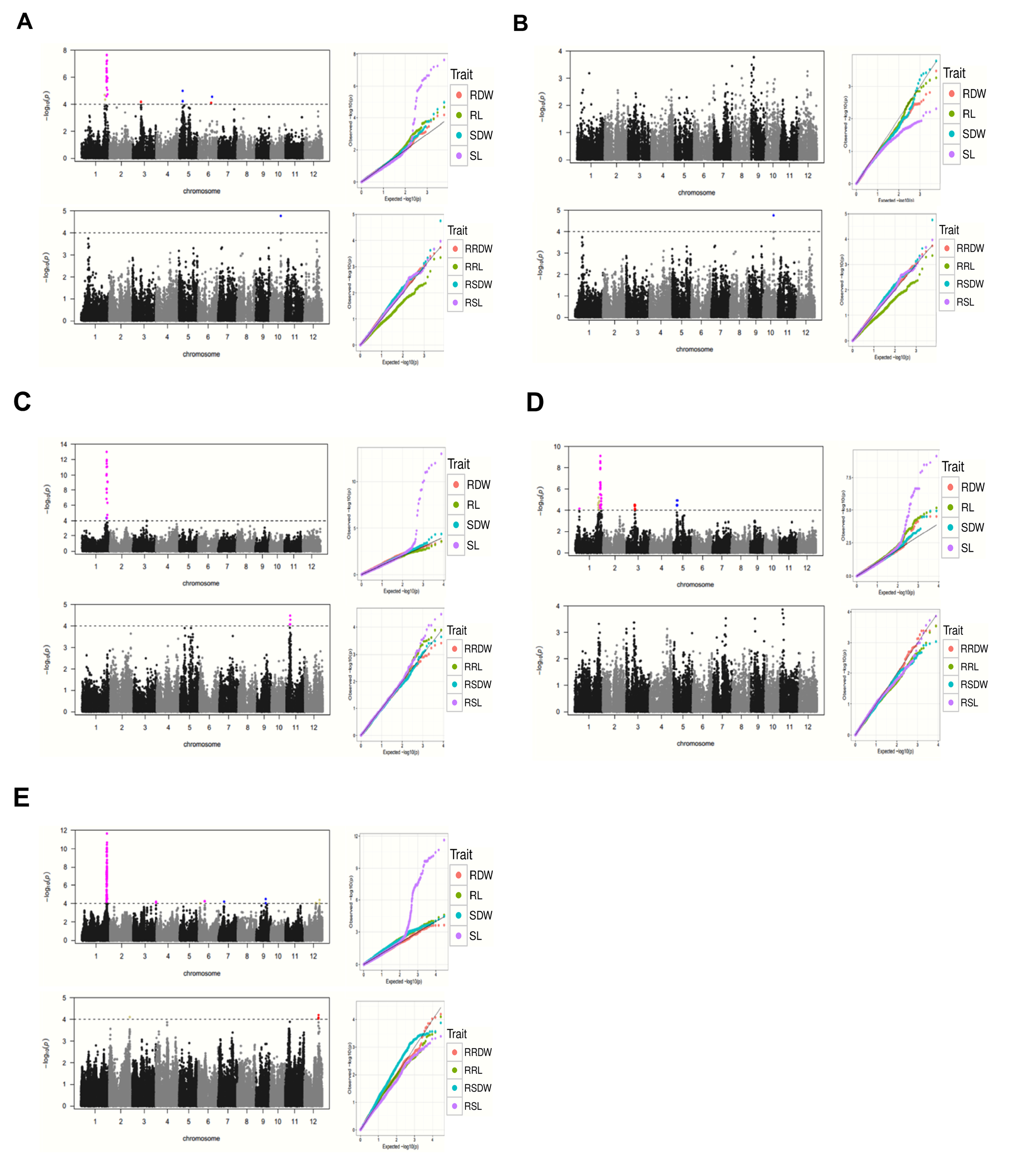

Supplement: Supplementary Figure S3 — Genome-wide association mapping for Al tolerance in DC1 (A), DC2 (B), 8way (C), DC12 (D), and RMPRIL (E) populations. Manhattan plots for Al tolerance at the seedling stage (red, khaki, blue, magenta, and green dots represent significant loci for traits RDW (RRDW), RL (RRL), SDW (RSDW), and SL (RSL) in Al stress condition, respectively) are on the left side of (A–E), horizontal short dash line on the Manhattan plot indicates the genome-wide significance threshold p ≤ 0.0001; Quantile-Quantile plots for Al tolerance are on the right side of (A–E). [file Image3.TIF]
