# Supplementary material for: Association Mapping of Ferrous, Zinc, and Aluminum Tolerance at the Seedling Stage in Indica Rice using MAGIC Populations
Source: Front Plant Sci. 2017 Oct 26;8:1822. doi: 10.3389/fpls.2017.01822 (PMC5662918; doi:10.3389/fpls.2017.01822)
Supplement: Supplementary Table S1 — Summary of the SNP selection procedure for the construction of the genetic map. [file Table1.docx]

**Supplementary TABLE S1 | Summary of the SNP selection procedure for the construction of the genetic map**

| Population | No. of lines | No. of SNP | Poly High Resolution | Filtered out monomorphic markers | Remove missing >10% | Remove MAF ^a^ <0.03 |
| --- | --- | --- | --- | --- | --- | --- |
| DC1 | 218 | 56607 | 39066 | 23507 | 22897 | 22160 |
| DC2 | 210 | 56607 | 39066 | 24482 | 23907 | 22020 |
| 8way | 445 | 56607 | 39066 | 30147 | 29409 | 28505 |
| DC12 | 428 | 56607 | 39066 | 30147 | 29360 | 28540 |
| RMPRIL | 873 | 56607 | 39066 | 30147 | 29371 | 28531 |

^a^ MAF: Minor allele frequency.
